# Switching gains and health plan price elasticities: 20 years of managed competition reforms in The Netherlands

**DOI:** 10.1007/s10198-017-0876-8

**Published:** 2017-02-27

**Authors:** Rudy Douven, Katalin Katona, Frederik T. Schut, Victoria Shestalova

**Affiliations:** 1 CPB Netherlands Bureau for Economic Policy Analysis, The Hague, The Netherlands; 2 Erasmus University Rotterdam, iBMG, Rotterdam, The Netherlands; 3 Dutch Healthcare Authority, Utrecht, The Netherlands; 4 VU Amsterdam, Amsterdam, The Netherlands; 5 Tilburg University, TILEC, Tilburg, The Netherlands

**Keywords:** Managed competition, Health insurance, Health plan price elasticity, Switching gains, I18, C33

## Abstract

In this paper we estimate health plan price elasticities and financial switching gains for consumers over a 20-year period in which managed competition was introduced in the Dutch health insurance market. The period is characterized by a major health insurance reform in 2006 to provide health insurers with more incentives and tools to compete, and to provide consumers with a more differentiated choice of products. Prior to the reform, in the period 1995–2005, we find a low number of switchers, between 2 and 4% a year, modest average total switching gains of 2 million euros per year and short-term health plan price elasticities ranging from −0.1 to −0.4. The major reform in 2006 resulted in an all-time high switching rate of 18%, total switching gains of 130 million euros, and a high short-term price elasticity of −5.7. During 2007–2015 switching rates returned to lower levels, between 4 and 8% per year, with total switching gains in the order of 40 million euros per year on average. Total switching gains could have been 10 times higher if all consumers had switched to one of the cheapest plans. We find short-term price elasticities ranging between −0.9 and −2.2. Our estimations suggest substantial consumer inertia throughout the entire period, as we find degrees of choice persistence ranging from about 0.8 to 0.9.

## Introduction

In health care systems with a competitive health insurance market sufficiently price-elastic, demand is important for motivating health insurers to act as cost-conscious purchasing agents on behalf of their customers. A recent systematic review of empirical studies on price elasticity of health plan choice identified clear-cut price elasticity ranges for different country settings but substantial variation in price elasticities across various countries [1]. For the Netherlands, where competition among health insurers was introduced within the social health insurance (SHI) scheme in 1996, the review study found short-term price elasticities smaller than −0.5, which were well below most of those found in other countries.^1^ As noticed by Pendzialek et al. [1], however, evidence about the Netherlands is dated, since the empirical studies only relate to the situation before a major health insurance reform in 2006, and almost no information could be found on price elasticities in the years following the reform. This limitation is particularly troublesome because the primary goal of the reform was to enhance consumer choice and competition in order to reinforce insurers’ incentives to improve the efficiency of care.

The main contribution of this paper is to fill this gap in the empirical literature by estimating the price elasticity of health plan choice in the Netherlands after the major reform in 2006. Using data on prices and market shares of all health plans over the period 2005–2015, we examine whether price elasticities of health plan choice increased relative to the low price elasticities prior to the reform. For a good comparison between the two periods, we re-estimated the price elasticities for the entire pre-reform period 1995–2005. This is because previous empirical studies use different methodologies and typically cover only part of the pre-reform period. As noticed by Pendzialek et al. [1], health plan price elasticities are difficult to compare because of the differences in methodologies and data sources of the included studies. Therefore, a second important contribution is that we provide consistent estimates of health plan price elasticities using the same methodology and data over a 20-year period. We are not aware of any other study that consistently estimated annual health plan choice over such a long period.^2^ Third, we contribute to the literature by also calculating the annual net financial switching gains for consumers over a 20-year period, uncovering also the sources of these gains. This provides a unique indication about the extent to which consumers financially benefited from switching and how these benefits changed over time. Therefore, our findings may offer important insights for health policy on how to influence consumer choice and price competition in health insurance markets.

Our paper is organized as follows. In “Overview of the Dutch health insurance market 1995–2015” we describe the main differences between the pre-reform and the post-reform health insurance market in the Netherlands. Section “Financial switching gains for premium payers” discusses the financial switching gains for premium payers. Section “Model and estimation methods” explains the estimation methods and empirical strategy. In “Data” we describe the data and in “Estimation results” the estimation results. Section “Conclusion” concludes.

## Overview of the Dutch health insurance market 1995–2015

### SHI market 1995–2005

In the past 20 years the Dutch health insurance system gradually moved towards a system of managed competition. Until 2005, health insurance for basic health care services consisted of a mandatory social health insurance (SHI) scheme for people in lower income brackets (about two thirds of the population) and a voluntary private health insurance system for people with a higher income.^3^ The SHI scheme was administered by sickness funds (not-for-profit health insurers). Health care expenses were largely covered by income-related contributions that were collected in a central fund and then redistributed to sickness funds. The share of income-related contributions as a percentage of total expenses was about 90% until 2002, and was reduced to about 80% in 2003. As a result, in 2003 the annual community-rated premium increased from about 10 to 20% of total expenses (see row “Out of pocket premiums/total cost (%)” in Table  1).^4^ To cover the residual costs, sickness funds were allowed to charge an annual community-rated premium (Table 2). Since 1993 sickness funds were increasingly put at risk for the medical expenses of their enrollees, by gradually replacing retrospective reimbursement by risk-adjusted capitation payments. In addition, the former legally protected regional monopolies were abolished and sickness funds were allowed to compete for customers all over the country. Eligible people were allowed to change sickness funds, and sickness funds were obliged to accept all applicants.^5^

**Table 1:** Characteristics of the population and health insurers in the Dutch SHI and HIA markets

Year	Social Health Insurance (about two thirds of population)											Health Insurance Act (total population)									
	1995	1996	1997	1998	1999	2000	2001	2002	2003	2004	2005	2006	2007	2008	2009	2010	2011	2012	2013	2014	2015
Population size of total market (million)^a^	9.7	9.8	9.9	9.9	9.9	10.3	10.3	10.2	10.1	10.2	10.1	16.3	16.4	16.3	16.4	16.5	16.6	16.7	16.7	16.8	16.8
Population of premium payers (million)^b^	7.7	7.7	7.9	8.0	8.1	8.3	8.3	8.2	8.2	8.2	8.2	12.5	12.6	12.8	12.9	13.0	13.1	13.2	13.3	13.3	13.4
Total number of health insurers^c^	26	27	29	29	29	26	24	21	21	21	21	33	32	32	30	28	27	26	26	26	25
Number of health insurers leaving/merging	1	0	0	2	0	3	2	3	0	0	0	n.a.	1	0	2	2	1	1	0	0	1
Number of health insurers entering	0	1	2	2	0	0	0	0	0	0	0	n.a.	0	0	0	0	0	0	0	0	0
Annual premiums/total cost (%)^d^	8	10	6	5	10	10	9	10	22	19	22	50	50	50	50	50	50	50	50	50	50
Insurers risk on medical expenses (%)^e^	3	13	27	28	35	36	38	41	52	53	53	53	53	52	60	73	70	88	90	92	99
Number of different insurance policies	26	27	29	29	29	26	24	21	21	21	21	46	53	60	56	57	56	60	65	67	71
Number of limited provider plans	0	0	0	0	0	0	0	0	0	0	0	0	0	1	1	5	5	8	10	12	17
Population share limited provider plans (%)	0	0	0	0	0	0	0	0	0	0	0	0	0	0.1	0.2	0.5	1.1	1.7	3.3	4.4	7.5
Population share with group contracts (%)^f^	0	0	0	0	0	0	0	0	0	0	0	53	57	60	60	64	66	67	69	70	69

If not otherwise indicated, the data was obtained from the Dutch Healthcare Authority [10, 33–38] and the Dutch National Healthcare Institute (ZIN)

^a^The privately insured are excluded from the population for 1995–2005

^b^The share of actual premium payers is about 80% of the total number of enrollees because insurance for children under 18 was free (financed by taxation)

^c^From 1995 to 2005 they were called sickness funds (not-for-profit health insurers). After 2006, for-profit insurers were also on the market

^d^Authors’ own calculations. As of 2006 the government mandated that 50% of the total cost should be paid in the form of annual premiums charged by the health insurer to the individual consumers (of 18 years and older)

^e^Insurance risk on medical expenses was obtained from Van Kleef et al. [39] and for 2013–2015 from personal communication with René van Vliet

^f^Before 2006 there were many group contracts in private insurance but not in the SHI market

**Table 2:** Annual premiums and switching characteristics in the Dutch SHI and HIA markets

Year	Social Health Insurance (about two-third of population)											Health Insurance Act (total population)									
	1995	1996	1997	1998	1999	2000	2001	2002	2003	2004	2005	2006	2007	2008	2009	2010	2011	2012	2013	2014	2015
Average annual premium (euros)^a^	89.8	155.7	98.2	97.9	178.8	189.7	163.6	182.6	344.7	304.6	378.1	1037	1115	1077	1080	1118	1214	1243	1232	1111	1168
Weighted premium, before switching (euros)^b^	89.9	155.4	97.7	97.7	178.7	187.7	157.0	181.5	355.7	307.5	384.8	1036	1105	1050	1059	1099	1201	1229	1217	1102	1163
Weighted premium, after switching (euros)^c^	89.9	155.4	97.7	97.7	178.6	187.5	156.8	180.4	355.2	307.0	384.0	1025	1103	1049	1059	1095	1199	1226	1213	1098	1158
Weighted premium individual contracts (euros)^d^	89.8	155.7	98.2	97.9	178.8	189.7	163.6	182.6	344.7	304.6	378.1	1056	1144	1091	1094	1137	1245	1275	1262	1137	1195
Weighted premium group contracts (euros)^d^	n.a.	n.a.	n.a.	n.a.	n.a.	n.a.	n.a.	n.a.	n.a.	n.a.	n.a.	998	1070	1019	1036	1070	1174	1201	1190	1080	1142
Standard deviation annual premiums (euros)	0	4	8	8	12	17	26	25	33	31	41	46	51	56	60	60	60	60	61	70	82
Number of switchers across insurers (%)	n.a.	n.a.	n.a.	n.a.	n.a.	n.a.	2.6	n.a.	2.8	2.4	4.2	17.8	4.4	3.6	3.6	4.3	5.5	6.0	8.2	7.0	7.3
Total switching gains (million euros)^e^	0.0	−0.1	0.2	0.4	0.8	1.6	1.3	1.2	4.3	3.7	6.7	130.0	31.8	26.6	2.6	51.1	45.1	44.6	49.2	53.0	53.8
Average switching gains premium payer (euros)^f^	0.0	0.0	0.0	0.1	0.1	0.2	0.2	0.1	0.5	0.5	0.8	10.4	2.5	2.1	0.2	3.9	3.5	3.4	3.7	4.0	4.0
Average gain per switcher (euros)^g^	n.a.	n.a.	n.a.	n.a.	n.a.	n.a.	5	n.a	15	15	16	45	44	45	4	72	50	45	36	45	44
Potential total switching gains (million euros)^h^	5	52	117	119	201	230	251	321	547	416	523	400	498	508	431	483	552	692	376	873	636
Potential average gain per premium payer (euros)^i^	1	5	12	12	20	22	24	31	54	41	51	32	39	40	33	37	42	52	28	65	47

If not otherwise indicated, the data was obtained from the Dutch Healthcare Authority [10, 33–38] and the Dutch National Healthcare Institute (ZIN)

HIA: The same calculation; however, first we calculated for each insurer the (weighted) average premium over all individual and group contracts

^a^Calculated as unweighted average annual premiums that each consumer pays to its health insurer (group and individual contracts taken together)

^b^Annual premiums weighted by insurers’ market shares in year *t*−1 (before switching) (group and individual contracts taken together, see also Appendix A)

^c^Annual premiums weighted by insurers’ market shares in year *t* (after switching) (group and individual contracts taken together, see also Appendix A)

^d^Calculated as weighted average premiums (weighted with health insurers’ market shares)

^e^Switching gains are calculated as the difference between the weighted premiums (after and before switching) multiplied by the number of premium payers in year *t* (see also Appendix A)

^f^Calculated as the total switching gains divided by the number of premium payers in the population (see Table 1)

^g^Calculated as the total switching gains divided by the number of switchers across insurers

^h^Calculated as the potential average gain per premium payer (last row) times the total population of premium payers

^i^SHI: calculated as the difference between the (weighted) average premium in the market in a year and a (weighted) average premium of the five cheapest insurers in the same year

As shown in Table 1, in 1996 the financial risk for the sickness funds was substantially raised (from 3 to 13%), resulting for the first time in meaningful differences in annual premiums.^6^ For this reason we chose 1995 as the starting year for estimating health plan price elasticities and switching gains. Incentives for price competition among sickness funds were gradually reinforced by stepwise increasing of sickness funds’ risk on medical expenses. This resulted in an increasing premium variation across sickness funds (Table 2).^7^

Next to premiums, sickness funds had only limited room to distinguish themselves. The room for negotiating different contracts with providers was almost nonexistent, since provider prices were highly regulated and selective contracting was only permitted for outpatient care. Moreover, sickness funds were not allowed to offer different health plans or to vertically integrate with providers. Five small sickness funds entered the market in the early years, but after 1998 only mergers took place and the number of sickness funds decreased from 26 to 21 in 2005 (Table 1 and Appendix B).

Sickness funds also provided supplementary health insurance, comprising about 5% of total revenues. Supplementary coverage typically includes dental care for adults, physiotherapy and medical appliances, such as spectacles and hearing aids. Supplementary and basic health insurance are often sold together so consumers might base their decisions to switch also on the combined premium [2].^8^

From 2001 to 2005 about 2–4% of the enrollees annually switched to another sickness fund [8]. Although the number of switchers from earlier years is lacking, the percentage of switchers from 1996 to 2000 is likely to be lower since the premium differences across sickness funds were small (Table 2) and many consumers might not even have been aware of the possibility of switching.^9^

### Health Insurance Act 2006–2015

In 2006 the scope of the managed competition model was broadened to the entire population by the introduction of a new Health Insurance Act (HIA). Former sickness funds and former private indemnity insurers were allowed to compete for providing basic health insurance to all Dutch citizens. The basic idea behind this reform was to increase efficiency by promoting more competition among health insurers and among health care providers. To preserve universal access and maintain equity the government followed a setup along the lines of the SHI, including mandatory insurance for a standardized basic benefit package, a partly community-rated and partly income-related premium, open enrollment and a risk adjustment system.

The government substantially increased health insurers’ financial risk by further abolishing ex-post cost reimbursement to health insurers. As shown in Table 1, for all health insurers the financial risk on medical expenses was gradually raised from 53% in 2005 to 99% in 2015.

In addition in 2006, the share of income-related premiums in total health care expenditure was reduced from 80% (SHI) to 50% (HIA), and this latter share is fixed by law (see row “Annual premiums/total cost (%)” in Table 1). This implied a significant increase of the annual premium for people previously enrolled in SHI from about 380 euros in 2005 to about 1050 euros in 2006 (see Table 2). The idea of policymakers behind this change was that a higher annual premium would make people more aware and cost-conscious of the high health care costs. To maintain equity, households with earnings below a certain threshold were compensated by monthly income-dependent subsidies.

Both sickness funds and private insurers were allowed to offer health insurance under the HIA. In 2006 basic health insurance was offered by 33 health insurers, but due to mergers and consolidation this number decreased to 25 in 2015, while no new insurers entered the market during this period (Table 1 and Appendix B).

In contrast to the former SHI scheme, the HIA offered health insurers several options to differentiate basic health insurance contracts to increase consumer choice. First, insurers were allowed to offer a voluntary deductible up to 500 euros per year in return for a premium discount. Next, health insurers were also allowed to offer group contracts at a premium discount of at maximum 10% of a similar individual contract. Third, health insurers were allowed to provide coverage in terms of service benefits, indemnity payments and a combination of both. Fourth, the HIA created more opportunities for health insurers to offer preferred or limited provider plans and to manage care by increasing the room for selective contracting and by allowing vertical integration with providers [9].

The introduction of the HIA had a large impact on the health insurance market. In the first year of the reform health insurers engaged in a premium war.^10^ Although people were not forced to switch health insurers since basic health plans were offered by both former sickness funds and private health insurers, for all people the choice setting and choice options radically changed. The massive media coverage around the reform, combined with a large increase in choice for different benefit packages and large premium differences, made many people aware of potential switching benefits. Hence, many people were triggered to reconsider their previous choice of health insurer. The threat of many customers making a cost-conscious choice forced insurers to offer contracts at annual premiums below the break-even price, resulting in substantial losses by insurers in 2006 [11]. This effectuated an all-time-high switching rate of 17.8% in 2006 (Table 2).^11^ Such a high switching rate was far above what had been experienced before in the Dutch health insurance markets (including the former private health insurance market). As shown in Table 2, during the first 4 years after 2006, switching rates between health insurers dropped from 18 to about 4%, but then increased to about 6–8% during the next 5 years.

Most health insurers offer both individual and group contracts. Group contracts can be concluded with any legal entity, and in total more than 50,000 group contracts are concluded annually with a huge variety of groups [10].^12^ Table 2 shows that group contracts are on average 50–70 euros per year lower priced than individual policies (implying a premium discount of about 5%). Most Dutch people have several options to join a group contract and the share of the population opting for a group contract increased from 53% in 2006 to 69% in 2015 (Table 1).

In addition to mandatory basic insurance, most people (about 85%) also bought voluntary supplementary insurance, just as in the former SHI market [10]. Although people can buy basic and supplementary coverage from different insurers, almost none did (only 0.19% of those buying supplementary insurance) [10]. As in the SHI market, the most important supplementary benefits are still dental care for adults and physiotherapy, but the variation in coverage substantially increased. Premiums for supplementary insurance plans are on average about 20–25% of those of basic health insurance plans [13]. Medical underwriting is allowed, but in practice only required for 5% of all supplementary policies (typically the most extensive ones) and for 24% of dental insurance policies [10]. Since almost all consumers buy supplementary and basic health insurance together, high-risk individuals may be restricted in choosing basic health plans by the underwriting practices of health insurers with respect to supplementary insurance. Indeed, several studies found that a substantial number of elderly and high-risk individuals do not switch to another insurer because they believe that they will not be accepted for supplementary insurance by another insurer [14, 15]. Boonen et al. [16] found that having supplementary insurance significantly reduces older people’s switching propensity.

Health insurers also compete with the premium discounts for people opting for a voluntary deductible on top of the mandatory deductible.^13^ The percentage of consumers choosing a voluntary deductible has increased from about 3% in 2006 to 12% in 2015 [13].

Since 2010 an increasing number of health insurers introduced lower-priced contracts with restricted provider networks and substantial co-payments for accessing outside network providers (Table 1). In 2015 about 7.5% of the population (1.25 million people) was enrolled in such a limited provider plan [10].

## Financial switching gains for premium payers

**Fig. 1:**
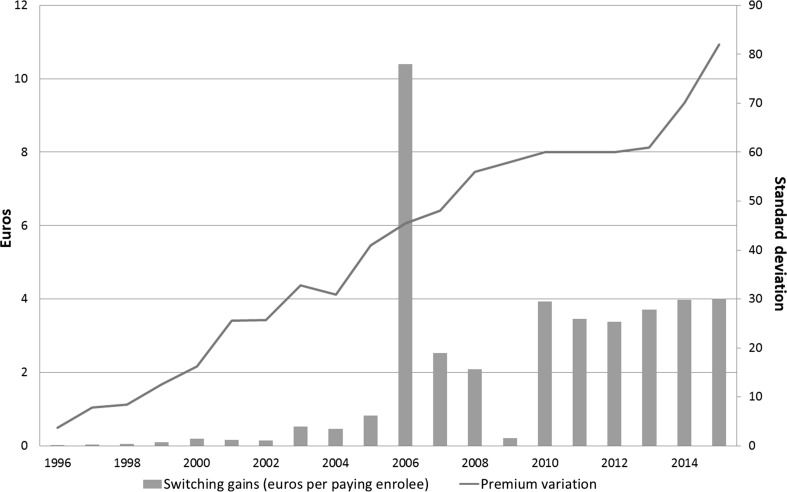
Average annual switching gains per premium payer and annual premium variation, 1996–2015. Switching gains per premium payer (see Table 2) are displayed on the *left axis* and premium variation (i.e. the standard deviation of annual premiums of group and individual contracts, see Table 2) on the *right axis*

Figure 1 exhibits switching gains and premium variation for the total 20-year period (1996-2015) making clear that switching gains for premium payers substantially increased due to the reform, with a peak in the reform year itself. We also observe an increasing trend in premium variation, corresponding with an increasing variety in health plan products after the reform year and the growing insurers’ risk on medical expenses. In the next subsections we discuss these switching gains.

### Switching gains in the SHI market (1996–2005)

To examine whether consumers respond to premium differences across health plans we calculated total annual switching gains. To that end we compare the total average annual premiums (weighted by insurers’ market shares) before and after switching (see Appendix A for a more detailed explanation). In Table 2 we show that total switching gains increased from zero in 1995 to 6.7 million euros in 2005. Thus, average total switching gains over the period are about 2 million euros per year. These switching gains are very modest. For example, in the year of the highest switching rate, 2005, average gains were 0.8 euros per premium payer and about 16 euros per switcher (i.e. 4% of the average annual premium). They are also very modest compared to potential total switching gains in the SHI market, which in any year could have been 100–200 times higher if all consumers had switched that year to one of the cheapest health insurers (see Table 2).^14^

There are several potential explanations for the observed increase in switching gains. First, only since 1996 have consumers been able to switch sickness funds once every year, and all sickness funds slowly started to compete on price and to attract customers from other sickness funds. Hence, it is likely that consumer awareness of switching opportunities has increased over time. Second, switching gains are likely to be larger when premium variation increases. Third, switching gains may also depend on institutional changes that affect insurers’ price setting behaviour. For instance, in 2003, several sickness funds had to raise their annual premiums because the government reduced the income-related contribution from 90 to 78% of total expenses. This change may have induced several sickness funds to adopt another pricing strategy. For example, large sickness funds were becoming relatively more expensive, which is reflected in Table 1 by the fact that for the first time weighted premiums before switching substantially exceeded the average premium. Notice, however, that in 2003 the weighted premiums after switching were also substantially higher than the average annual premiums, suggesting substantial consumer inertia, since many enrollees apparently decided to stick with the relatively expensive large sickness funds.

### Switching gains in the HIA market (2006–2015)

Calculating switching gains in the HIA market is more complicated than in the SHI market because consumers do not only switch between health plans but also between individual and group contracts of these health plans (for a detailed explanation, see Appendix A). The last two rows in Table 2 report the financial switching gains in this period. Switching gains were particularly high in the reform year 2006 with total switching gains of 130 million euros. The average gain per switcher remained fairly stable around 45 euros during 2006–2015 (see Table 2) but, compared to 2006, the number of switchers were substantially lower after the reform year.^15^ Still, with an average of 40 million per year during 2007–2015 total switching gains are quite modest, although much higher than prior to the reform. If in any year since 2007 all consumers had switched to one of the cheapest health insurers, total switching rates that year could have been about 10 times higher (see Table 2).

Nevertheless, consumers substantially benefited from switching since the introduction of the HIA. Table 3 shows a decomposition of the total switching gains into gains from switching within and between individual and group contracts. Initially, most switching gains came from switching from individual to group contracts, but as of 2011 this changed and most gains came from switching within individual contracts. In 2015, we observe for the first time a reverse trend and that more consumers switch from a group contract to an individual contract. This is likely to be the result of the introduction of cheaper individual contracts for health plans with limited provider networks in recent years that are targeted at young people, who are much more inclined to switch [17].

**Table 3:** Decomposition of switching gains in HIA market, 2006–2015 (in millions of euros)

	2008	2009	2010	2011	2012	2013	2014	2015
Total switching gains^a^	26.6	2.6	51.1	45.1	44.6	49.2	53.0	53.8
Within individual contracts	0.6	3.1	14.7	25.5	25.1	24.0	23.1	37.6
Within group contracts	−1.3	3.2	2.2	0.9	9.2	11.6	22.2	24.2
Shift from individual to group contract	27.3	2.7	34.2	18.7	10.3	13.6	7.7	−8.0

^a^Authors’ own calculations, see Appendix A

## Model and estimation methods

We estimate health plan price elasticities for three different periods: (1) prior to the reform 1995–2005, (2) the reform year 2006 and (3) the post-reform period 2007–2015.

For periods (1) and (3) we estimate an advanced dynamic model that follows from a standard discrete choice model, in which a consumer chooses an option out of all possible insurance policies in the market that maximizes his/her utility [18, 19]. The market share $s_{it}$ of each insurance policy $i$ in year *t* is represented by the multinomial logit equation:

1 $$s_{it} = \frac{{{ \exp }(\beta p_{it} + \gamma_{i} + \varepsilon_{it} )}}{{\mathop \sum \nolimits_{j} { \exp }(\beta p_{jt} + \gamma_{j} + \varepsilon_{jt} )}},$$

where *p*_*it*_ denotes the community-rated annual premiums. The health plan fixed effect $\gamma_{i}$ captures unobservable attributes that may differ across health plans, such as differences in the basic benefit package, health insurer quality, amount of advertising and the provision of supplementary insurance. Since data on these health plan attributes are not available we have to make the rather restrictive assumption that the impact of these attributes on market share do not change over time. We discuss the potential impact of this assumption on the estimation results in the Discussion Section. In addition, we assume that the stochastic term *ɛ*_*it*_ in the individual utility function is independent and has identically distributed extreme values [19, 20]. Taking logarithms and transforming this equation, we obtain:

2 $$\log \left( {s_{it} } \right) = \beta p_{it} + \gamma_{i} + \delta_{t} + \varepsilon_{it} ,$$

in which the term $\delta_{t}$ represents the denominator in Eq. (1). This model assumes that all consumers deliberately instantaneously choose a utility maximizing health insurance policy. Many researchers have already shown that this assumption does not hold for health insurance markets, which are characterized by a strong degree of persistence in health plan choice due to status quo bias, switching costs and information frictions [21–23]. To account for persistence in insurers’ market shares we follow Tamm et al. [18] and modify the equation by including a lagged market share term:

3 $$\log \left( {s_{it} } \right) = \alpha \log \left( {s_{i,t - 1} } \right) + \beta p_{it} + \gamma_{i} + \delta_{t} + \varepsilon_{it} ,$$

where 0 ≤ *α* ≤ 1 captures the average degree of persistence in the market. If *α* = 0 the model is static and in that case the model in Eq. (3) is similar to the instantaneous choice model in Eq. (2). If 0 < *α* < 1 there is some degree of persistence in the market that becomes larger when α is closer to one. From the specifications (1) and (3) we can derive the individual short-term and long-term premium elasticities, which we denote $\epsilon_{it}$ and *τ*_*it*_, and subsequently annual average price elasticities $\epsilon_{t}$ and *τ*_*t*_ that we will report in this study.^16^

4 $$\epsilon_{it} = \frac{{\partial s_{it} }}{{\partial p_{it} }}\frac{{p_{it} }}{{s_{it} }} = \beta p_{it} \left( {1 - s_{it} } \right),\; \epsilon_{t} \approx \beta \bar{p}_{t} ({\text{in case }} s_{it}\, {\text{is sufficiently small}})$$

5 $$\tau_{it} = \frac{1}{1 - \alpha }\epsilon_{it} ,\, \tau_{t} \approx \frac{1}{1 - \alpha }\epsilon_{t}$$

A property of the discrete choice model is that the elasticity in (4) is linearly related to the premium level *p*_*it*_, implying that health plans face a convex demand curve with regard to the level of the annual premium prevailing in the market. All else equal, if there is a linear relationship between price and elasticity, with the same coefficient *β*, the price elasticity is about 3 times higher in a market with a premium level of about 1000 euros (after the reform) than about 350 euros (prior to the reform, since 2003).

In “Estimation results” we will estimate specification (3) with an OLS-estimation and subsequently with generalized methods of moment (GMM) estimation. It is well known that estimating the dynamic specification (3) with standard fixed or random effect models is complicated since the lagged term log (*s*_*i*,*t*−1_) is likely to be correlated with the error term, the sum of *γ*_*i*_ and *ɛ*_*it*_. Under the assumption of serially uncorrelated errors of *ɛ*_*it*_ we can use a GMM estimator to obtain consistent estimates [24]. Premiums in (3) may also be endogenous. For example, setting a lower premium to attract new consumers may be less profitable for a large insurer because its loss on the incumbent enrollees is predictably higher than for a smaller insurer. GMM controls for this possible endogeneity of *p*_*it*_ by using lagged market shares and lagged premiums as instruments.

In the SHI market each insurer offers a single health plan with a similar benefit package. This is indicated in (1) by subscript *i*. However, in the HIA market, each insurer offers several health plans and often both individual and group contracts. We do have access to all individual contract prices in the market, but for group contracts we have only information about the total number of enrollees of all group contracts per health plan and the corresponding (weighted) average premium of these group contracts.^17^ In the HIA market the subscript *i* in (1) therefore refers to all individual contracts and a group contract with a weighted premium per health plan.

Finally, due to the integration of the former SHI and private insurance schemes into the HIA, we performed a separate estimation of health plan price elasticities during the year of the reform (period 2). The separate dataset for this transition covers 2 years, before (2005) and after (2006) the introduction of the HIA.

## Data

We obtained our data of health plan premiums and market shares from three different sources corresponding with the three periods, SHI, 1995–2005, the reform year 2006, and the HIA, 2007–2015. The first dataset was obtained from the Dutch National Health Care Institute (ZIN) and constitutes an unbalanced panel of 37 health plans (sickness funds) for 1995–2005 in the SHI (241 observations). In Appendix B, Table 7, we describe all sickness funds in the market.^18^

The second dataset was constructed by the Dutch Healthcare Authority (NZa) including 30 health insurers that were active in the years just before (2005) and just after the reform (2006) (see Appendix B, Table 8). For 2005 market shares in the voluntary private health insurance market were combined with market shares in the SHI market in order to construct a dataset that was comparable with HIA data on market shares (of both individual contracts and group contracts) and premiums in 2006 (in total 54 observations).

The third source is an unbalanced panel dataset of 26–32 health insurers for 2007–2015 in the HIA (in total 694 observations) that was also obtained from the NZa. Since health insurers were allowed to offer various health plans, we collected information on all “legally different” health plans, that is plans differing in terms of reimbursement method (in cash, in kind, or a combination of both) and contracted provider network. Next, we collected for each health insurer market shares for all individual contracts and an aggregated market share for all group contracts. Furthermore, we collected the corresponding annual premiums, and an average premium for all group contracts per insurer. For a description of the data, see Table 9 of Appendix B. Since many group contracts are not accessible for the entire population, aggregating all group contracts and using an average group premium per insurer is a simplification that may downward bias our estimates.^19^ However, aggregating all group contracts has the advantage that it suits our discrete choice model better, since a very large part of the population has the option to choose at least one group contract at the average premium, which would certainly not be the case if we considered each group contract separately in our estimations. This is because group contracts only differ in the price discounts offered by the insurer, and per insurer a group contract with an average discount rate is available to most individuals.^20^

## Estimation results

### Estimated health plan price elasticities for the SHI market (1996–2005)

As we explained in the Introduction, health plan price elasticities in the Dutch SHI market have been estimated before in several empirical papers. These studies found that estimated annual price elasticities were small and often below −0.5. However, these studies typically cover only part of the pre-reform period, use different estimation methods and did not include a lagged market share to control for persistence in health plan choice. Therefore, the price elasticities reported by these studies can be seen as short-term elasticities. By contrast, our study covers the entire period, and we estimate a dynamic model taking into account choice persistence which allows us to estimate long-term price elasticities as well. Especially in a longitudinal study over many years, it is important to control for changing market shares because these dynamic effects are not captured by fixed insurer effects.

Table 4 summarizes both OLS and GMM estimates of health plan price elasticities for the entire SHI period. We included the OLS estimates for a better comparison with the results for the reform year in which we could not use GMM because of the small dataset. In this particular case, the results of both methods appear to be close to each other.

**Table 4:** Estimation results for the health plan price elasticity in the SHI market 1996–2005

log (*s*_*it*_) = α log (*s*_*i*,*t*−1_) + *βp*_*it*_ + *γ*_*i*_ + *δ*_*t*_ + *ɛ*_*it*_, $\bar{p}_{t}$ between 100 and 400 euros (see also Table 1)		
(i) OLS estimation, number of observations: 243		
$\hat{\alpha }$ = 0.86^***^ (0.03)	$\hat{\beta } =$ −0.0011 (0.0006) $\hat{\varepsilon }_{t} \approx \hat{\beta }\bar{p}_{t}$ between −0.1 and −0.4 $\hat{\tau }_{t}$ between −0.8 and −3.1	*R* ^2^ = 0.97
(ii) System GMM estimation, number of observations used (including levels): 449		
$\hat{\alpha }$ = 0.91^***^ (0.02)	$\hat{\beta }= -$0.0011^***^ (0.0003) $\hat{\varepsilon }_{t} \approx \hat{\beta }\bar{p}_{t}$ between −0.1 and −0.4 $\hat{\tau }_{t}$ between −1.2 and −4.8	*R* ^2^ = 0.98

The estimations are performed with the plm-package in R [25], total number of insurance policies used is 37 (because a merged policy is treated as a new ID). Estimation (ii) includes individual effects. Sargan test: 36.1 (*D.f*. = 106, *P* value = 1), Wald test for coefficients (*D.f.* = 2) has a *P* value < 0.2 e−16. *R* ^2^ statistics is not a part of standard GMM output. It is added for the sake of comparison with the first regression in this table, defined as ${\text{corr}}\left( {s - \hat{s}} \right)^{2}$. Additional estimations results are available by the authors upon request

* *P* value <0.1; ** *P* value <0.05; *** *P* value <0.01

The OLS estimates correspond to short-term price elasticities ranging between −0.1 and −0.4 depending on the premium level. This range is consistent with the results of previous studies. For the GMM estimations we included time dummies, individual effects and reported robust standard errors. We report the GMM system estimator with endogenous premiums [26], which we prefer for the following reasons. First, according to the econometric literature, the system GMM estimator has a better performance in terms of bias and efficiency than the first-difference GMM estimator. Second, premiums are likely to be endogenous both because market shares may be associated with market power and because large health plans may be less willing to reduce premiums (e.g. because of solvency regulations). Based on Sargan statistics [27, 28], we cannot reject the hypothesis that the over identifying restrictions of the system GMM estimator are valid.

The short-term price elasticity resulting from the GMM estimator in estimation (ii) ranges from −0.1 (at a base premium of 100 euros) to −0.4 (at a base premium level of 400 euros). This estimate implies that a health insurer increasing its annual premium by 1% (about 1–4 euros) would cause an insurer’s market share to decline by about 0.1–0.4%, depending on the premium level. The size of these price elasticities is similar to the OLS estimates and those found in previous studies.

We found a high degree of persistence in the SHI market of around 90%, implying that most enrollees were sticking with a once chosen sickness fund. Strong persistence implies that long-term price elasticities are much higher than short-term price elasticities. According to our discrete choice model, Eq. 5, this strong persistence implies that long-term price elasticities range from −0.8 to −4.8. This means that if an insurer increases its premium by 1% each year, for an infinite number of years, then its market share would decline by 0.8–4.8%. The long-term elasticities are extremely sensitive to the precise estimation of the degree of persistence.

### Estimated health plan price elasticities for the reform year 2006

For estimating health plan price elasticities in the reform year a specific dataset was constructed, comprising only 2 years (2005 and 2006). Given the small dataset we can only use OLS to estimate Eq. (3) without fixed effects and time dummies. Table 5 summarizes the estimations results.

**Table 5:** Estimation results for the health plan price elasticity in the reform year 2006

log (*s*_*it*_) = α log (*s*_*i*,*t*−1_) + *βp*_*it*_ + *ɛ*_*it*_, $\bar{p}_{t} = 1025$ euros		
OLS estimation, reform year *t* = 2006, number of observations: 54		
$\hat{\alpha }$ = 0.84^***^ (0.05)	$\hat{\beta}= -$ 0.0055^***^ (0.0021), $\hat{\varepsilon }_{t} \approx \hat{\beta }\bar{p}_{t} = - 5.7$ (2.1) $\hat{\tau }_{t}$ = −35.6	*R* ^2^ = 0.84

We have fewer observations in our estimations than insurance policies in the data because new insurers entering the market in 2005 have a market share of zero and drop out of the sample

* *P* value <0.1; ** *P* value <0.05; *** *P* value <0.01

The results indicate a high degree of choice persistence of 84% in the market.

This result is in line with our earlier observation that a large part of the population did not switch from health insurer. We found a high health plan price elasticity of about −5.7, which corresponds to the all-time high number of switchers of 18% in 2006. The estimated price elasticity implies that an average health insurer increasing its annual premium by 1% (about 10 euros) would, in 2006, experience a decline in market share of about 5.7%. Nevertheless, even in the reform year most people did not switch health plans, despite health plan annual premiums for all people changing dramatically relative to the preceding year. As a matter of fact, we still found a high degree of persistence of 84%, implying a corresponding extremely high long-term price elasticity of −35.6 for the reform year 2006.

A limitation of the estimated price elasticity is that because of the short period we could not include fixed effects to account for insurer specific characteristics (e.g. differences in supplementary insurance, service quality and rebates for voluntary deductibles). However, surveys among consumers indicate that, especially in 2006, price was the most important determinant of health insurer choice [12].

### Estimated health plan price elasticities for the HIA market (2007–2015)

Table 6 summarizes the estimation results for the health plan price elasticity during the post-reform period. As for the SHI market we present the results of both the OLS estimation (including only time dummies) and GMM estimation (including time dummies and individual effects), and we report the GMM system estimator with endogenous premiums [26].

**Table 6:** Estimation results for the health plan price elasticity in the HIA market 2006–2015

log (*s*_*it*_) = α log (*s*_*i*,*t*−1_) + *βp*_*it*_ + *γ*_*i*_ + *δ*_*t*_ + *ɛ*_*it*_, $\bar{p}_{t}$ ranging from 1100 to 1300 euros (Table 2)		
(iv) OLS estimation, Number of observations: 577		
$\hat{\alpha }$ = 0.95^***^ (0.01)	$\hat{\beta }= -$0.0017^**^ (0.0008), $\bar{\varepsilon }_{t} = \hat{\beta }\bar{p}_{t}$ between −1.9 and −2.2 $\hat{\tau }_{t}$ between −34 and −40	*R* ^2^ = 0.97
(*v*) GMM estimation, number of observations (including levels): 1013		
$\hat{\alpha }$ = 0.81^***^ (0.03)	$\hat{\beta }= -$ 0.0008^***^ (0.0001), $\bar{\varepsilon }_{t} = \hat{\beta }\bar{p}_{t}$ between −0.9 and −1.0 $\hat{\tau }_{t}$ between −4.7 and −5.5	*R* ^2^ = 0.99

The estimations are performed with the plm-package in R [25], total number of policies used is 155, making distinction between collective and individual policies and using only policies with a minimum of 10,000 enrollees. We use a system GMM estimator with endogenous premiums, including time dummies and fixed effects. Sargan statistics (85.83, *D.f.* = 68, *P* value = 0.071), Wald test for coefficients of this model (*D.f.* = 2) has a *P* value <0.2 e−16. Additional estimations results are available by the authors upon request

* *P* value <0.1; ** *P* value <0.05; *** *P* value <0.01

As shown in Table 6, estimated short-term price elasticities range between −0.9 and −2.2 depending on the estimation method. This is higher than in the pre-reform SHI market, but substantially lower than in the reform year. Compared to the previous period, we find a larger discrepancy between the OLS and GMM estimates. This can arise due to both a wider variety and greater fluctuation in the number of health plans offered in the market in this period (as shown in Table 2), which increase premium endogeneity affecting OLS estimates. Yet, we report the OLS results as an upper bound, since robustness checks using alternative GMM specifications resulted in price elasticities higher than 0.8 in absolute value.^21^

The results in Table 6 show that consumer inertia in the HIA market is almost as high as in the SHI market, with a degree of persistence of 80–90%. The degree of choice persistence does not substantially differ between the three estimation periods, but is significantly different from one. Long-term price elasticities range from −5 to −40 and are again very sensitive to the estimation of the degree of persistence.

It is possible that our long-term price elasticities are overstated because the calculations assume that the short-term price elasticity remains constant over the years. However, it is likely that some consumers are more persistent in their choice of health plan than others, and that in practice we only observe a limited group of potential switchers that are price sensitive. Comparing our findings with Tamm et al. [18] for the German social health insurance market, the only other study that measured the degree of persistence in the same way, we find lower long-term price elasticities. Tamm et al. [18] cannot reject a degree of persistence of 100% (*α* = 1) implying that long-term price elasticties are infinite, indicating that market shares of German health insurers could follow a random walk.

A conceptual problem with the interpretation of elasticities is that a high degree of persistence may indicate the presence of perfect competition or high consumer inertia. In the case of perfect competition there would be no consumer mobility since consumers would have chosen the optimal insurance product and insurers would fully adjust their prices to changes in marginal costs over the years. As shown in many studies, however, status quo bias and consumer inertia play a large role in health insurance markets [21, 29]. Our findings show that the Dutch health insurance market is no exception. From 2006 to 2014, 69% of the Dutch population never switched to another health insurer despite a growing premium variation [30].^22^

### Limitations

Although we estimated the health plan price elasticities using the same methodology and a similar dataset, a good comparison of health plan price elasticities between the SHI and HIA periods is still complicated.

A limitation of our study is that by using fixed effects we can control for constant differences of unobserved market and institutional characteristics, but we cannot control for possible changes in these characteristics. A first market characteristic that may change over time and for which we lack sufficient data is supplementary insurance.^23^ Since about 85% of the population buys supplementary insurance and almost all from the same insurer from which they obtain basic health insurance, changes in supplementary insurance may have affected basic health plan choice. In contrast to supplementary insurance, basic health insurance offers are very transparent and easy to compare, because benefit packages are the same across all insurers, premiums are community-rated and cost-sharing arrangements are standardized (deductible levels are the same across all basic health plans). Therefore, most health insurers primarily use the basic health plan premiums in their marketing activities during the open enrollment period at the end of each year. Comparing prices and benefit packages of supplementary insurance is much more complicated for consumers because products and prices are difficult to compare and because of the vast number of supplementary insurance packages that are offered.^24^ Since having supplementary insurance is found to be negatively related to people’s propensity to switch basic health plans [16], the increasing differentiation of supplementary insurance products may have resulted in downward-biased price elasticities of basic health plan choice in later years.

A second market characteristic that has changed since 2006 is the proportion of people opting for a voluntary deductible for basic health insurance.^25^ People of 18 years and older can choose a voluntary deductible of 100, 200, 300, 400 or 500 euros on top of the mandatory deductible in return for a premium discount.^26^ The number of people choosing a voluntary deductible has increased from about 6% in 2006 to about 12% in 2015 [13]. If there is no strong correlation across insurers between health plan premiums with or without a voluntary deductible, our price elasticities are likely to be biased. However, we find that premiums of both types of health plans are highly correlated, and therefore this bias may not be substantial.^27^

A third changing characteristic of the HIA market is the increasing role of limited-provider plans, making health plans more heterogeneous over time. As shown in Table 1 the share of the population enrolled in limited provider plans gradually increased from 0.1% in 2008 to 7.5% in 2015. Limited provider plans are typically lower priced than health plans with unrestricted provider choice. If limited provider plans are offering lower (perceived) quality than unrestricted health plans, our price elasticities may be biased downwards because it is likely that more people would have chosen lower-priced plans if quality would have been the same.

A comparison of price elasticities between SHI and HIA is also complicated by the changes in institutional characteristics of the choice setting. An important difference between the SHI and HIA is the way in which premium subsidies are structured. As a result premium levels in the HIA market are on average 3 to 6 times higher than in the SHI market. Adjusting for the different premium levels would reduce the difference between the estimated price elasticities between both markets, but it is not clear to what extent because the impact of the premium level on price elasticity is difficult to assess. For instance, different premium levels may induce a different behavioural response, since consumers may not only respond to *absolute* but also to *relative* premium differences [31]. All other things equal, this would result in higher price elasticities in the SHI market than in the HIA market.^28^ Price elasticities may also be somewhat higher in SHI than HIA because the SHI did not cover high-income people, who may be less sensitive to price than lower-income people (because of a diminishing marginal utility of money).

Another difference that may complicate a good comparison is the much higher number of choice options in the HIA market than in the SHI market. This may have had a downward effect on the price elasticity in the HIA market because of information and choice overload [32].

Since it is not possible to disentangle all these possible effects, we cannot determine to what extent the increase in estimated health plan price elasticities was driven by the reform. For this reason the quantitative changes in health plan price elasticities we estimated should be interpreted only as a rough indication of the impact of 20 years of managed competition reforms in the Netherlands.

## Conclusion

In 1996 managed competition was introduced in the Dutch social health insurance (SHI) scheme. From 1996 to 2005 health insurers had few tools and limited incentives to compete, and consumers had little incentive to switch. In 2006 a major reform was implemented to provide insurers with more incentives and tools to compete and to provide consumers with a more differentiated health plan choice. Using data on prices and market shares of all health plans over a 20-year period (1995–2015) we provide a long-term overview with respect to the number of switchers, switching gains and health plan price elasticities in the Dutch insurance market. The Dutch setting is especially interesting because it describes the first and subsequent steps of introducing managed competition into a social health insurance market. This information is not only useful for Dutch policymakers but also for other countries following a similar path.

Prior to the reform (1995–2005) we find modest increasing total switching gains increasing from about 0 in 1995 to 7 million euros per year in 2005. The reasons are small premium variations and a low number of switchers, between 2 to 4% a year. If all consumers had switched to one of the lowest priced health plans, switching gains could have been 100–200 times larger in any year (of course this holds only for a single year and not for the entire period). We find modest short-term health plan price elasticities ranging from −0.1 to −0.4, depending on the annual premium level.

The introduction of the reforms (2006) resulted in an all-time high switching rate of 18% and a health plan price elasticity of −5.7. Moreover, switching gains for consumers peaked with total gains of 130 million in the first year of the reform (2006). The main reason is that the reform had a large impact on consumer awareness of switching possibilities.

In the post-reform decade (2007–2015), the number of switchers returned to lower levels with the proportion of switchers increasing from about 4 to 8%. Consumers financially benefited much more from switching health plans than in the SHI (on average about 45 euros per switcher per year since 2006), although total switching gains in any year still could have been about 10 times higher if all people had switched to one of the lowest priced health plans. We find health plan price elasticities that range from −0.9 to −2.2.

A good comparison of short- and long-term health plan price elasticities between the SHI and HIA period is complicated because in our estimations we may not perfectly control for unobserved changing market and institutional characteristics. We do find strong evidence of substantial consumer inertia as the degree of choice persistence varied from about 0.8 to 0.9 during the 20-year period. Strong persistence also implies that long-term price elasticities could be much higher than short-term price elasticities, because people only slowly respond to changing prices.

The high forgone potential switching gains and high level of persistence suggest that many people make suboptimal choices, particularly because quality differences between health plans appear to be small [10]. Therefore, an active policy to improve health plan choice may be welfare enhancing in this case. One option is to increase transparency in the insurance market by facilitating better informed consumer choices.^29^ Although comparative health plan information is readily available on the internet, this information is often incomplete (e.g. lacking information on available group contracts) and sometimes biased by commercial interests (e.g. brokers’ fees are paid when the consumer enrolls into a health plan via a comparison website). It is important to ensure that choice sites offer independent, complete and comprehensive information on health plans (and if possible also on group contracts). Also, consumer education campaigns on how to choose a suitable health plan and how to recognize a good quality choice site is a way of improving consumer choice. Another option for lowering consumer search costs is to improve the choice structure for the type of health plans offered by insurers (perhaps ‘bronze’, ‘silver’, ‘gold’ and ‘platinum’ health plans, which would be easy to distinguish for consumers). In the Netherlands, some steps have been taken in this direction by requiring insurers to publish prices of “similar” health plans that are sold under different labels or through different channels. It is expected that this will make it easier for consumers to select the cheapest plan. Similar rules could also be imposed with respect to information on premium discounts on group contracts, many of which are open to all consumers. Lastly, insurers could be obliged to inform consumers actively, regarding changes in the contracted provider network relevant to the consumers’ residential area.

## Appendix A

In this appendix we explain how we calculated switching gains. In the SHI market, 1996-2005, this is straightforward and switching gains SG_*t*_ in year *t* are defined as:

$${\text{SG}}_{t} = N_{t} \mathop \sum \limits_{j} P_{j,t} (s_{j,t - 1} - s_{j,t} )$$

*P*_*j*,*t*_ is the premium of insurance policy of insurer *j* in year *t*, *s*_*j*,*t*_ is the market share of insurer *j* in year *t* and *N*_*t*_ is the total number of premium payers in year *t*.

In the HIA market it is more complicated because we have individual and group contracts. Most of these policies were offered both as individual contracts and as group contracts. Our dataset is complete for all years, except for 2006 and 2007.^30^ When an insurance firm withdraws some policy from the market, it usually reallocates the enrollees to the closest alternative policy in its portfolio, and this alternative policy becomes in our calculations the default option of these enrollees for the next year.^31^ In the computation of switching gains we make the following two assumptions. First, if an insurer offers a policy in year (*t*−1) but does not offer the same policy in year *t* then we assume that in year *t* these enrollees would be offered the closest available policy of the same insurer as a ‘default option’. We use this assumption for individual and group contracts. Next, we allocate the individual and group market shares of policies that exited the market in year (*t*−1) to the respective default options. Secondly, if a new policy enters the market in year *t* (which is not a default option of a policy leaving the market) then we assume the market share of this policy in year (*t*−1) was zero. The total switching gains represent the weighted average price change that arises because of reallocation of enrollees among contracts, multiplied by the number of premium payers *N*_*t*_:

$${\text{SG}}_{t} = N_{t} \mathop \sum \limits_{j} P_{j,t}^{i} (s_{j,t - 1}^{i} - s_{j,t}^{i} ) + P_{j,t}^{c} (s_{j,t - 1}^{c} - s_{j,t}^{c} ),$$

where *P*_*j*,*t*_^*i*^ is the premium of all individual insurance policies of insurer *j* in year *t*, *P*_*j*,*t*_^*c*^ is the premium of all group (or collective) insurance policies of insurer *j* in year *t*, *s*_*j*,*t*_^*i*^ is the market share of all individual insurance policies of insurer *j* in year *t* and *s*_*j*,*t*_^*c*^ is the market share of all group insurance policies of insurer *j* in year *t.*

These gains can be attributed to three sources:

1. Switching gains within the individual insurance segment: *SG*_*t*_^*i*^
2. Switching gains within the group insurance segment: ${\text{SG}}_{t}^{c}$
3. Switching gains from individual to group (or vice versa): *SG*_*t*_^*ic*^

We can calculate these three types of switching gains. First we define

$$\Delta P_{t}^{i} = \mathop \sum \limits_{j} P_{j,t}^{i} (s_{j,t - 1}^{i} /s_{t - 1}^{i} - s_{j,t}^{i} /s_{t}^{i} )$$

$$\Delta P_{t}^{c} = \mathop \sum \limits_{j} P_{j,t}^{c} (s_{j,t - 1}^{c} /s_{t - 1}^{c} - s_{j,t}^{c} /s_{t}^{c} )$$

$$\Delta P_{t}^{ic} = \mathop \sum \limits_{j} P_{j,t}^{i} s_{j,t}^{i} /s_{t}^{i} - P_{j,t}^{c} s_{j,t}^{c} /s_{t}^{c}$$

where $s_{t}^{i}$ and $s_{t}^{c}$ are market shares of the respective segment at time *t*. Note that the market shares sum up to one at any time *t*: ∑_*j*_ *s*_*j*,*t*_^*i*^ + *s*_*j*,*t*_^*c*^ = *s*_*t*_^*i*^ + *s*_*t*_^*c*^ = 1, and $P_{j,t}^{i}$ and *P*_*j*,*t*_^*c*^ denote individual and collective premiums of policy *j* at time *t*. Using these notations, the total switching gains are decomposed into the three sources as follows:

$${\text{SG}}_{t} = {\text{SG}}_{t}^{i} + {\text{SG}}_{t}^{c} + {\text{SG}}_{t}^{ic} = \Delta P_{t}^{i} s_{t - 1}^{i} N_{t - 1} + \Delta P_{t}^{c} s_{t - 1}^{c} N_{t - 1} + \Delta P_{t}^{ic} (s_{t}^{c} - s_{t - 1}^{c} )N_{t - 1}$$

## Appendix B

See Tables 7, 8 and 9.

**Table 7:** Health insurers that were active in the SHI market during 1995–2005

	Health insurer	Operating years	Number of annual obs.	Additional information^a^
1	AGIS	2001–2005	5	New large insurer, merger of Anova, ZAO, ANOZ
2	Amicon	1995–2005	11	Large insurer
3	Anderzorg	1995–2005	11	Small insurer
4	Anova	1995–2001	7	Large regional insurer, merged in 2002 into AGIS
5	ANOZ	1996–2001	6	Large regional insurer, merged in 2002 into AGIS
6	Azivo	1995–2005	11	Medium sized regional insurer.
7	CZ Groep	1995–2005	11	Large insurer
8	De Friesland	1995–2005	11	Medium sized regional insurer
9	DSW	1995–2005	11	Medium sized regional insurer
10	Geove	1995–2005	11	Medium sized insurer
11	Groene Land	1995–2005	11	Large insurer
12	Nederzorg	1998–2005	8	New small insurer
13	Nuts	1995–2005	11	Medium sized insurer
14	NZC	1997–1999	3	New small insurer, left market in 2000
15	OHRA	1995–1999	5	Small insurer, merged with Nuts in 2000
16	ONVZ	1997–2005	9	New small insurer
17	OZ	1996–2005	11	Large insurer
18	OZB	1998–2005	8	New small insurer
19	Pro Life	1996–2000	4	New small insurer, merged in 2001 with ANOVA
20	PWZ	1995–2001	7	Medium sized insurer, merged in 2002 with Groene Land
21	Salland	1995–2005	11	Small insurer
22	SR Rotterdam	1995–2005	11	Small regional insurer
23	Topzorg	1995–1999	4	Small regional insurer, merged with Geove in 2000
24	Trias	1995–2005	11	Medium sized regional insurer
25	Univé	1995–2005	11	Large insurer
26	VGZ	1995–2005	11	Large insurer
27	ZAO	1995–2001	7	Large regional insurer, merged in 2002 into AGIS
28	ZK	1995–2005	11	Large insurer
29	ZK Noordwijk	1995–1997	3	Medium sized regional insurer, merged in 1998 with ZK
30	ZK Spaarneland	1995–1997	3	Medium sized regional insurer, merged in 1998 with ZK
31	ZON	1995–1999	5	Medium sized insurer, merged in 2000 with Amicon
32	Zorg and Zekerheid	1995–2005	11	Medium sized regional insurer

^a^Our observation series are unbroken and cover 100% of the market. In total we obtained 270 observations. Note that in our estimations we need at least three consecutive years of data to perform GMM estimations. An insurer is denoted “small” if in the last year of the sample the market share was less than 1%, “medium sized” if the market share was between 1 and 5%, and “large” if the market share was larger than 5%. We also indicate whether an insurer operated mainly regionally

**Table 8:** Health insurers that were active during the reform years 2005–2006

	Health insurer	Operating years	Number of annual obs.^a^	Additional information^b^
1	AGIS	2005–2006	2	Large insurer, operated in 2005 in SHI and PHI market
2	Anderzorg	2005–2006	2	Small Insurer, operated in 2005 in SHI and PHI market
3	Avéro	2005–2006	2	Medium sized insurer, operated in 2005 in PHI market
4	Azivo	2005–2006	2	Small insurer, operated in 2005 in SHI market
5	AZVZ	2005–2006	2	Small insurer, operated in 2005 in PHI market
6	Confior	2005–2006	2	Small insurer, operated in 2005 in PHI market
7	CZ–Group	2005–2006	2	Large insurer, operated in 2005 in SHI and PHI market
8	De Friesland	2005–2006	2	Medium sized insurer, operated in 2005 in SHI and PHI market
9	De Goudse	2005–2006	2	Small insurer, operated in 2005 in PHI market
10	Delta Loyd	2005–2006	2	Medium sized insurer, operated in 2005 in PHI market
11	DSW	2005–2006	2	Medium sized insurer, operated in 2005 in SHI market
12	FBTO	2005–2006	2	Medium sized insurer, operated in 2005 in PHI market
13	FORTIS	2005–2006	2	Medium sized insurer, operated in 2005 in PHI market
14	Groene Land	2005–2006	2	Large insurer, operated in 2005 in SHI market
15	Interpolis	2005–2006	2	Small insurer, operated in 2005 in PHI market
16	IZA	2005–2006	2	Medium sized insurer, operated in 2005 in PHI market
17	IZZ	2005–2006	2	Medium sized insurer, operated in 2005 in PHI market
18	Menzis	2005–2006	2	Large insurer, operated in 2005 in SHI and PHI market
19	OHRA	2005–2006	2	Medium sized insurer, operated in 2005 in PHI market
20	ONVZ	2005–2006	2	Medium sized insurer, operated in 2005 in SHI market
21	OZ	2005–2006	2	Medium sized insurer, operated in 2005 in SHI market
22	OZB	2005–2006	2	Small insurer, operated in 2005 in SHI and PHI market
23	PNO	2005–2006	2	Small insurer, operated in 2005 in PHI market
24	Salland	2005–2006	2	Small insurer, operated in 2005 in SHI market
25	SR	2005–2006	2	Small insurer, operated in 2005 in SHI market
26	Trias	2005–2006	2	Medium sized insurer, operated in 2005 in SHI and PHI market
27	UMC	2005–2006	2	Small insurer, operated in 2005 in PHI market
28	Univé	2005–2006	2	Medium sized insurer, operated in 2005 in SHI and PHI market
29	VGZ	2005–2006	2	Large insurer, operated in 2005 in SHI and PHI market
30	Zorg and Zekerheid	2005–2006	2	Medium sized insurer, operated in 2005 in SHI and PHI market
31	ZK	2005–2006	2	Large insurer, operated in 2005 in SHI and PHI market

^a^Prior to 2006 some health insurers were only active in the social health insurance (SHI) market, some only in the private health insurance market (PHI) and some in both markets. Since 2006 all insurers have been active in the same market, but market shares of all insurers had to be collected from both markets in 2005. For each health insurer we obtained market shares of all individual contracts and (the sum) of all group contracts in 2005. For 2006 we obtained individual and group market shares and corresponding nominal premiums, where the premium for the group contracts for each insurer is calculated by taking the average (with market share) weighted premiums of all individual group contracts

^b^We denoted whether an insurer was active in 2005 as a former sickness fund in the SHI market and/or as a private indemnity insurer on the PHI market. An insurer is denoted “small” if, in the year 2006, its market share was less than 1%, “medium sized” if the market share was between 1 and 5%, and “large” if the market share was larger than 5%

**Table 9:** Health insurers that operated in the HIA market during 2007–2015

	Health insurer	Holding 2016^c^	Operating years	No. of Annual obs.	No. of policies^a^	Additional information^b^
1	AGIS	Achmea	2007–2014	8	2–5	Large insurer, since 2008 part of holding Achmea
2	Anderzorg	Menzis	2007–2015	8	1	Medium sized insurer
3	ASR	ASR	2007–2015	8	2–4	Medium sized insurer
4	Avéro	Achmea	2007–2011	8	2–4	Large insurer
5	Azivo	Menzis	2007–2015	8	1	Medium sized insurer, since 2008 part of holding Menzis
6	Confior	Menzis	2007–2008	2	2	Small insurer, merged in 2009 into Menzis
7	De Friesland	Achmea	2007–2015	8	2–5	Large insurer, since 2012 part of holding Achmea
8	Delta Lloyd	CZ	2007–2015	8	1–2	Medium sized insurer, since 2008 part of holding CZ
9	Eno	Eno	2007–2015	8	1–2	Medium sized insurer
10	FBTO	Achmea	2007–2015	8	1–2	Medium sized insurer
11	Groene land	Achmea	2007–2009	3	1–2	Large insurer, merged in 2010 into Zilveren Kruis
12	Interpolis	Achmea	2007–2015	8	1	Medium sized insurer
13	IZA	VGZ	2007–2015	8	1–3	Large insurer
14	IZZ	VGZ	2007–2015	8	1–2	Medium sized insurer
15	Menzis	Menzis	2007–2015	8	2–4	Large insurer
16	Univé	VGZ	2007–2015	8	3–5	Large insurer
17	Cares	VGZ	2007–2015	8	2–3	Small insurer
18	UMC	VGZ	2007–2015	8	1	Medium sized insurer
19	OHRA	CZ	2007–2015	8	1–3	Medium sized insurer, since 2008 part of holding CZ
20	OHRA Zorg	CZ	2007–2015	8	1–4	Medium sized insurer, since 2008 part of holding CZ
21	ONVZ	ONVZ	2007–2015	8	1	Medium sized insurer
22	AZVZ	Z&Z	2007–2010	4	1	Small insurer, exit in 2011, taken over by holding Z&Z
23	CZ	CZ	2007–2015	8	2–4	Large insurer
24	DSW	DSW–SH	2007–2015	8	1	Medium sized insurer
25	Z&Z	Z&Z	2007–2015	8	2–3	Medium sized insurer
26	OZF	Achmea	2007–2015	8	1	Small insurer
27	PNO	ONVZ	2007–2009	3	1	Small insurer, exit in 2010, taken over by holding ONVZ
28	Stad Holland	DSW–SH	2007–2015	8	1	Small insurer
29	Trias	VGZ	2007–2011	5	2	Medium sized insurer, since 2012 part of holding VGZ
30	Univé Zorg	VGZ	2007–2008	2	2	Medium sized insurer
31	VGZ	VGZ	2007–2015	8	1–4	Large insurer
32	Zilveren Kruis	VGZ	2007–2015	8	2–11	Large insurer

^a^The Table reports the number of different health plans existing within 1 year. The number of different health plans offered by the same insurer may fluctuate over the years. These health plans could be sold either via individual contracts or group contracts or both. In the period studied, most health plans were sold via both individual and group contracts

^b^An insurer is denoted “small” if its market share was smaller than 100,000, “medium sized” if the market share was between 100,000 and 500,000, and “large” if the market share was larger than 500,000 enrollees

^c^Many health insurers are operating within a larger holding company as separate legal entities. Often the name of the holding company is the same as the name of the largest health insurer within the holding

## Footnotes

1 Short-term health plan price elasticities in the SHI market have been estimated before in several empirical studies, covering the years before 2000 [2, 3], the period until 2002 [4] and the same SHI period as in this paper [5].

2 In the systematic review by Pendzialek et al. [1] most of the 41 included empirical studies cover only a few years. Only three studies cover more than 10 years but include various institutional settings over the years.

3 The SHI scheme can be regarded as the precursor of the HIA scheme introduced in 2006 because both schemes have many features in common (i.e. both are mandatory insurance schemes with a comprehensive standardized benefits package, partly income-related and partly community-rated premiums, and carried out by competing health insurers). Therefore, we compare the SHI with HIA here and do not consider the voluntary private health insurance scheme. Private indemnity health insurance covered about a third of the Dutch population with earnings above a legally determined income threshold. Benefit packages were similar to that of SHI, although there was substantial variation in both the scope of benefits and cost-sharing arrangements. Enrollment was voluntary, premiums were risk-rated and medical underwriting was allowed. For an extensive description of the private insurance market, see Tapay and Colombo [6], and for the SHI market, see Douven and Schut [7].

4 Income-related contributions were annually set by the government as a percentage of gross income up to an annually adjusted threshold (about 32,600 euros in 2005).

5 From 1993 to 1995 people were allowed to switch once every 2 years. To facilitate consumer choice, since 1996 fixed annual open enrollment periods were introduced.

6 From 1993 to 1995, except for one small sickness fund, all sickness funds charged the same annual premium.

7 An increase in price competition may also result in lower premium variation. However, since premium competition was absent before 1996 we interpret the increase in premium variation as a sign of increasing price competition.

8 Supplementary benefit packages were quite similar across health insurers. Schut and Hassink [2] found a somewhat higher price elasticity for combined health insurance (−0.4) than basic health insurance (−0.3).

9 In 2001, 83% of the sickness fund enrollees responded in a survey that they had not even considered switching. This share declined to 77% in 2005 [8].

10 Health insurers have to announce new health plan premiums each year before November 20 and enrollees that are willing to switch have to notify their insurer before the end of the year that they want to terminate the contract. Every year since 2006 the same small regional insurer is the first to announce its premium early in October, attracting a lot of free publicity. Until 2015 most health insurers announced new health plan premiums 10–20 days before the deadline, but in 2015 about half of the insurers waited until the last week and the variation in announcement dates decreased [10]. The health insurer with the lowest health plan premium typically waits until all other health plan premiums are known, in order to be sure of being the cheapest health plan.

11 In addition to the 18% of the population switching between health insurers, 5-10% changed health plans within their insurer, so including these intra-insurer switchers raises the total number of switchers in 2006 to 23–28% [12].

12 In 2015, 56% of group contracts were employer-based, while other group contracts were concluded with a large variety of entities, such as labor unions, sport federations, cooperative banks, and interest associations for elderly and patients (e.g. for diabetes and rheumatoid arthritis) [10]. Group contracts with elderly and patient organizations are feasible because health insurers are compensated for predictably high expenditures by the risk adjustment system.

13 Initially, in 2006 and 2007, there was a mandatory no-claim rebate of 255 euros per year. In 2008, this no-claim rebate was replaced by a mandatory deductible of 150 euros per year, which was gradually raised to 375 euros per year in 2015.

14 Notice that (potential) switching gains and premium setting are interrelated. It is likely that an increase in the number of switchers would result in more premium competition, which would reduce premium variation and potential switching gains.

15 The low switching gains per premium payer in 2009 (see Table 2) are for a large part explained by one insurer who raised its premium on a large group contract to above the average premium in the market, while only a few consumers in this group contract switched to a lower priced contract [13]. In general, participating in a group contract is associated with a lower switching propensity [16].

16 For a more extensive discussion of the derivation and properties of the elasticities, see Tamm et al. [18] and Train [19]. Average annual elasticities are calculated by a weighted average of the insurer specific elasticities. In our study we have many health insurance policies (or health plans) allowing us to make the simplifying assumption $\varepsilon_{t} \approx \beta \bar{p}_{t}$.

17 This information is collected through insurer surveys by the Dutch healthcare authority.

18 The panel is unbalanced because of mergers. After a merger the merging insurers were removed from the dataset and a new merged insurer was added to our dataset in the year before the merger.

19 Price elasticities are estimated under the assumption that consumers have free choice. Since not all group contracts are equally accessible this will downward bias our estimate for the price elasticity.

20 A distinction can be made between employer-based group contracts and other group contracts. Employer-based group contracts are typically only accessible for employees and dependents of the specific employers. Most other group contracts are open group contracts, meaning that they are accessible for all people joining the legal entity concluding the contract. In 2015 the average premium discount of employer-based group contracts relative to the same individual contract was 8.5%, whereas the average discount on the other group contracts was 6.4% [10].

21 Not shown here, but available from the authors upon request.

22 Individual level data from almost all health insurers show that 20% of the population switched once, 7% twice, 2% three times and 1% switched four times between 2006 and 2015 [30].

23 There is some fragmented information available on supplementary benefits packages and premiums for a few recent years but consistent time series are lacking. Schut and Hassink [2] tackled the problem of differentiated supplementary insurance products by using the price of a ‘most common’ supplementary benefits package and by re-estimating their equations with the total price (i.e. the sum of basic and supplementary insurance premiums). However, their study covered only the period 1996–1998 in the SHI market when product differentiation was limited.

24 In 2015 people could choose among 276 supplementary health plans, of which 94 were specific dental health plans, resulting in more than 1300 possible choice combinations [10].

25 Taking into account voluntary deductibles complicates the estimation procedure because only a few consumers opt for such a deductible.

26 The mandatory deductible was 150 euros in 2007 and it was gradually raised in subsequent years to 375 euros in 2015.

27 The Pearson correlation between the full premium and the discounted premium ranges from 0.81 to 0.98 in 2008-2015, whereas the Spearman rank correlation ranges from 0.63 to 0.88 over the same period. In 2007, both correlations were somewhat lower (about 0.5) but in that year the share of the enrollees choosing a voluntary deductible was extremely low.

28 This is an interesting topic for future research.

29 As shown by Handel [29], the welfare effects of improving health plan choice are theoretically ambiguous because the positive welfare effects may be offset by increasing adverse selection. In the Dutch case, however, adverse selection is effectively constrained by sophisticated risk adjustment. Therefore, it is likely that in the Dutch setting improving individual-level plan choices will enhance welfare.
